# PD-L1 expression in Xp11.2 translocation renal cell carcinoma: Indicator of tumor aggressiveness

**DOI:** 10.1038/s41598-017-02005-7

**Published:** 2017-05-18

**Authors:** Kun Chang, Yuanyuan Qu, Bo Dai, Jian-Yuan Zhao, Hualei Gan, Guohai Shi, Yiping Zhu, Yijun Shen, Yao Zhu, Hailiang Zhang, Dingwei Ye

**Affiliations:** 1 Department of Urology, Fudan University Shanghai Cancer Center, Shanghai, 200032 China; 2 Department of Oncology, Shanghai Medical College, Fudan University, Shanghai, 200032 China; 3 Department of Pathology, Fudan University Shanghai Cancer Center, Shanghai, 200032 China; 4 The State Key Laboratory of Genetic Engineering and Collaborative Innovation Center of Genetics & Development, School of Life Sciences, Fudan University, Shanghai, 200433 China

## Abstract

Programmed death ligand-1 (PD-L1), a promising antitumor target, has proven clinical value against many malignancies. However, the PD-L1 content of Xp11.2 translocation renal cell carcinoma (Xp11.2 RCC) and its correlation with clinical outcomes remain unclear. This study aimed to investigate PD-L1 expression in Xp11.2 RCC and to assess its prognostic value. Formalin-fixed paraffin-embedded specimens from 36 adult patients that were histologically confirmed (by fluorescence *in situ* hybridization) were subjected to immunohistochemical analysis. Of the 36 Xp11.2 RCC patients, 9 (25.0%) had tumors with positive PD-L1 expression and 27 (75.0%) had tumors with negative PD-L1 expression. Positive PD-L1 expression correlated with advanced tumor stage (P = 0.001), regional lymph node metastasis (P < 0.001), and distant metastasis (P < 0.001). A multivariate analysis identified positive PD-L1 expression was an independent adverse prognostic factor for both progression free survival (hazard ratio: 3.7, P = 0.018) and overall survival (hazard ratio: 4.5, P = 0.034). The median PFS and OS for the whole cohort were 13.0 months (95% confidence interval [CI], 9.4–16.6 months) and 36.0 months (95% CI, 23.9–48.1 months), respectively. Our findings suggest that positive PD-L1 expression is indicative of worse clinical outcome in Xp11.2 RCC. Further studies are needed to explore the potential efficacy of targeting PD-L1 in Xp11.2 RCC.

## Introduction

Renal cell carcinoma (RCC) is widely recognized as a heterogeneous disease with various histological subtypes. The most common histopathological subtypes are clear cell (60%–75%), papillary (10%–15%), chromophobe (5%), and collecting duct carcinomas^1^. Xp11.2 translocation RCC (Xp11.2 RCC) is a rare subtype that was recognized as a distinctive pathological entity in the 2004 World Health Organization renal tumor classification^2, 3^. Xp11.2 RCC is characterized by several translocations on chromosome Xp11.2, resulting in gene fusion between TFE3 and at least six possible partners^4–6^.

As it is a rare RCC subtype, the best treatment for Xp11.2 RCC has not been defined. Surgery is the optimal treatment for localized Xp11.2 RCC patients, including those with positive regional lymph nodes^7^. However, previous studies indicate that Xp11.2 RCC presents at an advanced stage with a rapid clinical course^8, 9^. As a result, systematic treatment may be indispensable for most patients. Anti-VEGF drugs are reported to have activity against metastatic Xp11.2 RCC^10, 11^. However, Xp11.2 RCC has poor prognosis regardless of treatment^12, 13^. Therefore, new, effective treatments are desperately needed for patients with this tumor type.

Monoclonal antibodies (mAbs) targeting the programmed death 1 (PD1)/programmed death ligand-1 (PD-L1) pathway have achieved impressive response rates in patients with melanoma, non-small cell lung cancer, and bladder cancer, and PD-L1 has been validated as a predictive biomarker for the outcome of mAb therapy in many studies^14–16^. However, its prognostic and clinical value in patients with Xp11.2 RCC subtypes is unknown. In this study, we sought to investigate the levels of PD-L1 expression and its correlation with clinical outcome in a series of patients with Xp11.2 RCC that was histologically confirmed using TFE3 break-apart fluorescence *in situ* hybridization (FISH).

## Results

### Patient characteristics

**Figure 1:**
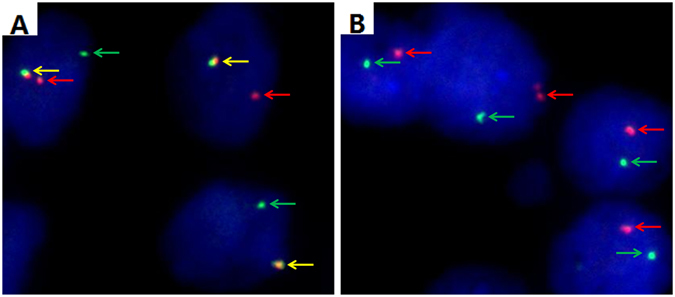
Representative images of the TFE3 break-apart probe assay. (**A**) Separate red and green signals (indicated by the respective arrows) and normal co-hybridization signals (yellow arrows) indicate that one X chromosome harbors the translocation and the other is normal in a female patient (×1000). (**B**) Separate red and green signals (indicated by the respective arrows) indicate that the only X chromosome harbors the translocation in a male patient (×1000); TFE3, transcription factor E3.

Representative images of the TFE3 break-apart FISH assay show the classical TFE3 rearrangement associated with Xp11.2 translocation (Fig. 1 **)**. The clinicopathological features of the patient cohort are summarized in Table 1. Of the 36 Xp11.2 RCC patients, 13 were male (36%) and 23 were female (64%), with a median age of 29 years (range, 14–63). The median follow-up was 30 months (range, 2–87 months). At the last follow-up, 11 patients (31%) had died of Xp11.2 RCC and 11 (31%) patients had progressive disease. The median PFS and OS for the whole cohort were 13.0 months (95% confidence interval [CI], 9.4–16.6 months) and 36.0 months (95% CI, 23.9–48.1 months), respectively.

**Table 1:** Clinicopathological characteristics in relation to PD-L1 expression status.

**Variable**	Entire group (n = 36)	PD-L1 expression		*P* value
		Negative expression (n = 27)	Positive expression (n = 9)	
Median age at surgery (y, range)	29.0 (14.0–63.0)	30.0 (14.0–63.0)	23.0 (14.0–47.0)	0.375
Sex (n, %)				0.161
Male	13 (36)	8 (30)	5 (56)	
Female	23 (64)	19 (70)	4 (44)	
Clinical manifestation (n, %)				0.845
Incidental	15 (42)	11 (41)	4 (44)	
Symptomatic	21 (58)	16 (59)	5 (56)	
Laterality (n, %)				0.700
Left	18 (50)	14 (52)	4 (44)	
Right	18 (50)	13 (48)	5 (56)	
Surgical option (n, %)				0.121
Radical nephrectomy	30 (83)	21 (78)	9 (100)	
Partial nephrectomy	6 (17)	6 (22)	0 (0)	
Median tumor size (cm, range)	5.7 (2.0–18.0)	6.0 (2.0–11.2)	5.1 (2.3–18.0)	0.783
T stage at presentation (n, %)				**0.001**
T1-T2	24 (67)	22 (82)	2 (22)	
T3-T4	12 (33)	5 (18)	7 (78)	
N stage at presentation (n, %)				** < 0.001**
N0	22 (61)	21 (78)	1 (11)	
N1	14 (39)	6 (22)	8 (89)	
M stage at presentation (n, %)				**<0.001**
M0	28 (78)	25 (93)	3 (33)	
M1	8 (22)	2 (7)	6 (67)	
ISUP grade (n, %)				0.355
2	8 (22)	5 (18)	3 (33)	
3–4	28 (78)	22 (82)	6 (67)	
Adjuvant therapy (n, %)				0.125
None	13 (36.1)	11 (84.6%)	2 (15.4%)	
Immunotherapy	5 (13.9)	5 (100.0%)	0 (0.0%)	
Targeted therapy	18 (50.0)	11 (61.1%)	7 (38.9%)	

### PD-L1 expression in Xp11.2 RCC

**Figure 2:**
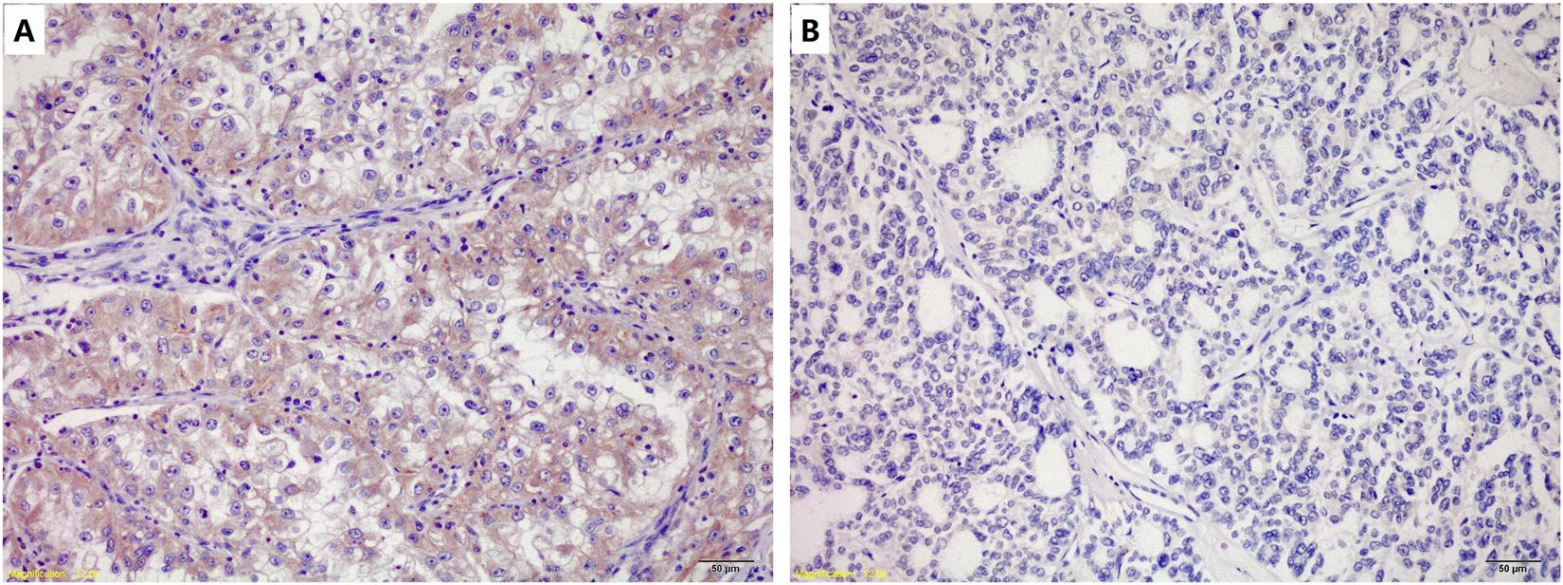
Immunohistochemical analysis of programmed death receptor 1 (PD-L1) expression in Xp11.2 RCC. (**A,B**) Tumor sections with (**A**) positive, and (**B**) negative PD-L1 expression (magnification, ×200). Note that PD-L1 protein is expressed on the cell membrane as well as the in the cytoplasm of tumor cells.

PD-L1 expression was mainly confined to the tumor cell membrane, with or without cytoplasmic expression. Tumor samples of 9 Xp11.2 RCC patients (25%) had positive PD-L1 expression and 27 (75%) had negative PD-L1 expression (representative images shown in Fig. 2).

### Correlations between PD-L1 expression level and patient characteristics

PD-L1 expression levels and clinicopathological parameters are listed in Table 1. PD-L1 expression status was not associated with age (*P* = 0.375), sex (P = 0.161), clinical manifestation (*P* = 0.845), tumor laterality (*P* = 0.700), surgical option (*P* = 0.121), tumor size (*P* = 0.783), and ISUP grade (*P* = 0.355). There was significant correlation between positive PD-L1 expression and a higher tumor stage (*P* = 0.001). Approximately 80% of TNM T_3–4_ stage tumors, but only 22% of T_1–2_ stage tumors had positive PD-L1 expression. Positive PD-L1 expression was more common in tumors with regional lymph node metastasis (89%) than in N_0_ stage tumors (11%, *P* < 0.001) and in the tumors of patients presenting with distant metastasis than in those with localized disease (*P* < 0.001).

### Clinical outcome according to PD-L1 expression

**Figure 3:**
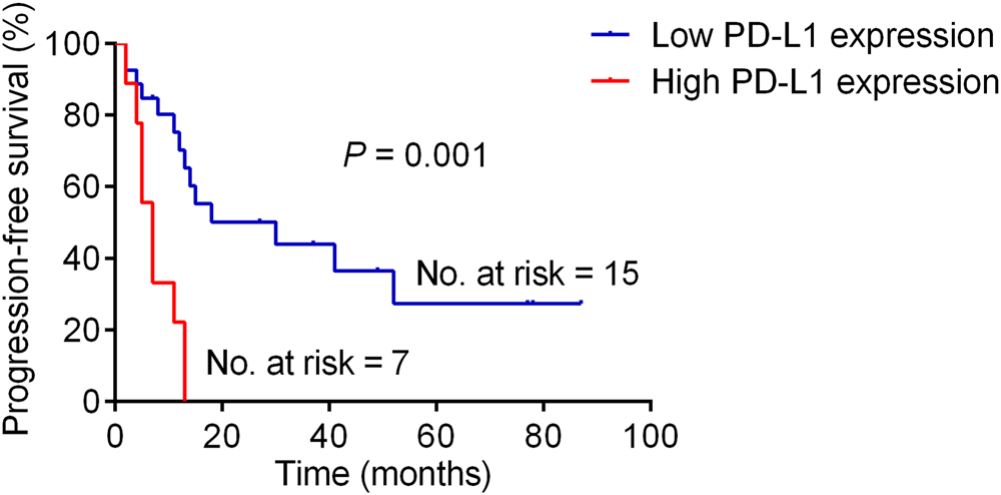
Kaplan–Meier analysis of progression-free survival in the patient cohort stratified by PD-L1 status.

**Figure 4:**
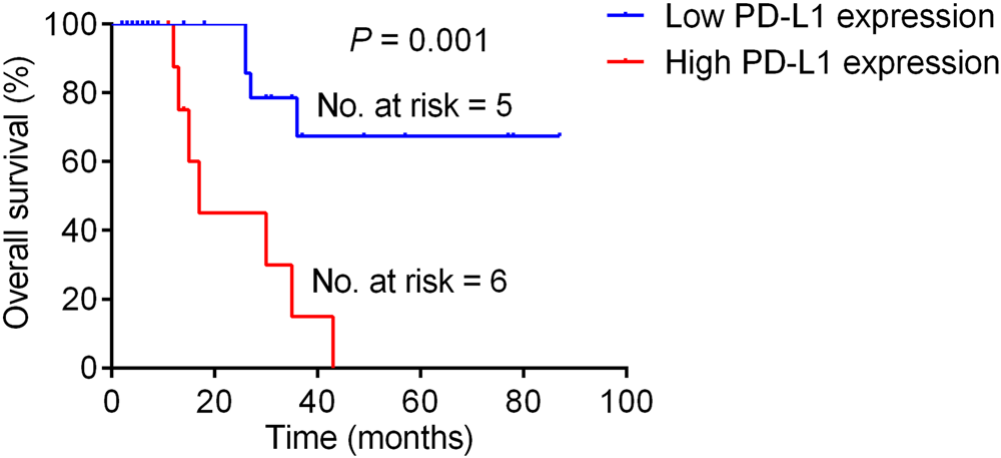
Kaplan–Meier analysis of overall survival in the patient cohort stratified by PD-L1 status.

The PFS was shorter for patients who had tumors with positive PD-L1 expression (median time to disease progression, 7 months) than for those with for tumors with negative PD-L1 expression (median time to disease progression, 30 months; P = 0.01; Fig. 3). Positive tumor PD-L1 expression was significantly associated with shorter OS (*P* = 0.001): 78% (7/9) of patients with positive tumor PD-L1 expression had a median OS of 17 months (95% CI, 12.1–21.9), whereas only 15% (4/27) of those with negative tumor PD-L1 expression died of the disease within the study period (median OS, not reached; Fig. 4 **)**.

### Potential prognostic factors affecting PFS and OS

The prognostic value of each clinicopathological factor, including PD-L1 expression status, was evaluated for PFS (Table 2) and OS (Table 3). Univariate Cox proportion hazard ratio analysis showed that tumor T stage (hazard ratio [HR]: 3.8, *P* = 0.005), N stage (HR: 5.6, *P* = 0.001), M stage (HR: 13.7, *P* < 0.001), and PD-L1 expression status (HR: 4.7, *P* = 0.003) were associated with PFS. Further, tumor N stage (HR: 4.0, *P* = 0.025), M stage (HR: 9.6, *P* = 0.004), and PD-L1 expression status (HR: 3.7, *P* = 0.018) were identified as independent prognostic factors of PFS by multivariate analysis. Advanced tumor T stage (HR: 7.3, *P* = 0.007), regional lymph node metastasis (HR: 10.6, *P* = 0.003), distant metastasis (HR: 17.2, *P* < 0.001), ISUP grade (HR: 0.2, *P* = 0.030) and positive PD-L1 expression (HR: 6.7, *P* = 0.003) were associated with shorter OS. After adjusting for all the clinical and pathological parameters tested in this study, only distant metastasis (HR: 11.0, *P* = 0.027) and positive PD-L1 expression (HR: 4.5, *P* = 0.034) were independently associated with OS.

**Table 2:** Univariate and multivariate Cox regression analyses of PFS in 36 enrolled adult Xp11.2 RCC patients.

Covariates	Univariate analysis		Multivariate analysis	
	HR (95% CI)	P value	HR (95% CI)	P value
Age at surgery	1.0 (0.9–1.0)	0.068		
Sex (male vs. female)	0.6 (0.3–1.5)	0.297		
Clinical manifestation (incidental vs. symptomatic)	0.9 (0.4–2.2)	0.888		
Laterality (left vs. right)	1.2 (0.5–2.7)	0.725		
Surgical option (radical vs. partical)	0.4 (0.1–1.6)	0.186		
Tumor size	1.1 (0.9–1.2)	0.324		
T stage (T1-T2 vs. T3-T4)	**3.8 (1.5–9.8)**	**0.005**	2.5 (0.8–7.6)	0.097
N stage (N0 vs. N1)	**5.6 (2.0–15.5)**	**0.001**	**4.0 (1.2–13.7)**	**0.025**
M stage (M0 vs. M1)	**13.7 (3.5–54.4)**	**<0.001**	**9.6 (2.0–45.0)**	**0.004**
ISUP grade (2 vs. 3 or 4)	0.6 (0.2–1.9)	0.375		
PD-L1 expression (negative vs. positive)	**4.7 (1.7–13.0)**	**0.003**	**3.7 (1.3–10.9)**	**0.018**

PFS, Progression-free survival; Xp11.2 RCC, Xp11.2 translocation renal cell carcinoma.

**Table 3:** Univariate and multivariate Cox regression analyses of OS in 36 enrolled adult Xp11.2 RCC patients.

Covariates	Univariate analysis		Multivariate analysis	
	HR (95% CI)	P value	HR (95% CI)	P value
Age at surgery	1.0 (0.9–1.0)	0.268		
Sex (male vs. female)	0.6 (0.2–1.9)	0.353		
Clinical manifestation (incidental vs. symptomatic)	0.7 (0.2–2.4)	0.606		
Laterality (left vs. right)	0.8 (0.2–2.6)	0.670		
Surgical option (radical vs. partical)	0.0 (0.0–22.8)	0.309		
Tumor size	1.0 (0.9–1.2)	0.855		
T stage (T1-T2 vs. T3-T4)	**7.3 (1.7–30.4)**	**0.007**	3.4 (0.8–14.7)	0.107
N stage (N0 vs. N1)	**10.6 (2.3–49.8)**	**0.003**	5.7 (0.8–41.6)	0.086
M stage (M0 vs. M1)	**17.2 (3.3–87.9)**	**<0.001**	**11.0 (1.3–91.2)**	**0.027**
ISUP grade (2 vs. 3 or 4)	**0.2 (0.1–0.9)**	**0.030**	0.3 (0.1–1.7)	0.173
PD-L1 expression (negative vs. positive)	**6.7 (1.9–23.4)**	**0.003**	**4.5 (1.1–17.7)**	**0.034**

OS, overall survival; Xp11.2 RCC, Xp11.2 translocation renal cell carcinoma.

## Discussion

PD-L1 (also termed B7-H1 or CD274) is highly expressed in a number of tumor types and is recognized as a strong prognostic factor for affected patients. PD-L1 binding to its receptor, PD-1, on activated T lymphocytes and other immune cells negatively regulates T-cell proliferation and activity. This interaction allows tumor cells to evade immune surveillance and eradication. Many malignancies with positive PD-L1 expression was associated with poor patient prognosis. This is especially so for cancers originating in the epithelium, such as esophageal cancer, gastric cancer, and oropharyngeal squamous cell carcinoma^17^.

Thompson *et al*. were among the first to identify the clinical significance of PD-L1 expression in clear cell RCC (ccRCC) patients using immunohistochemical methods^18–20^. These authors found that PD-L1-positive tumors were more likely to have adverse pathological features including a late tumor stage (III or IV), high International Society of Urological Pathology (ISUP) grade (III or IV), larger tumor size, and a higher risk of cancer-specific mortality for patients. Krambeck *et al*. reported that survivin expression status combined with PD-L1 expression status gave a more accurate prognosis for ccRCC patients. High survivin expression combined with PD-L1 positivity was significantly associated with a high risk of death for ccRCC patients in both univariate (risk ratio, 12.82; 95% CI, 7.50–21.92; *P* < 0.001) and multivariate (risk ratio, 2.81; 95% CI, 1.56–5.04; *P* < 0.001) analyses. In addition to immunohistochemical analyses, circulating PD-L1 expression has also been examined in patient peripheral blood samples. In Frigola’s study, preoperative circulating PD-L1 levels in ccRCC patients were quantified by ELISA^21^. Higher preoperative PD-L1 levels in blood samples were associated with larger tumors and those with an advanced stage, higher grade, and necrosis. A doubling of soluble PD-L1 levels was associated with a 41% increased risk of death. For the metastatic ccRCC patients treated with VEGF-targeted therapies, Toni K *et al*.’s study also suggested that increased tumor cell PD-L1, or PD-L1 plus tumor CD8-positive T-cell counts, were associated with shorter survival^22^. As a result, accumulating evidence indicates that elevated PD-L1 is a negative predictor for survival in ccRCC patients.

So far, only one study has attempted to measure PD-L1 expression in Xp11.2 RCCs. Choueiri *et al*. reported that out of 10 Xp11.2 RCC patients, 3 had positive PD-L1 expression in tumor cells and 9 harbored PD-L1-positive tumor infiltrating immune cells^23^. However, the prognostic value of these findings and their correlation with clinical variables were not analyzed due to limited patient numbers. In our study, from 2246 consecutive patients, 36 Xp11.2 RCC patients were enrolled which were histologically confirmed by TFE3 break-apart FISH analysis. We demonstrated that positive PD-L1 expression was considerablely associated with advanced T stage (T_3–4_), regional lymph nodes or distant metastasis. Additionally, positive tumor PD-L1 expression indicated a worse prognosis for Xp11.2 RCC patients.

Recently, PD-1/PD-L1-targeted immunotherapy has been shown to be clinically effective and thus a promising therapeutic option for several malignancies. Clinical trials have also suggested that clinical response rates strongly correlate with PD-L1 expression status. This was first demonstrated in a phase I study of nivolumab in solid tumors (CA209-003)^24^. Of 46 patients with melanoma, NSCLC, or RCC, 25 tumors were positive and 21 were negative for PD-L1. Tumor PD-L1 expression was evaluated immunohistochemically. A treatment response was seen in nine PD-L1-positive patients (36%) but in none of the PD-L1-negative patients. The authors also noted that multiple tumor sections should be analyzed and that a patient was considered PD-L1 positive if at least one biopsy sample was positive. Powles *et al*. investigated the atezolizumab responsive of bladder cancer patients stratified by PD-L1 expression status^25^. The response rate was 43% for patients with PD-L1-positive tumors, but only 11% for patients with PD-L1-negative tumors. Recently, McDermott *et al*. reported that atezolizumab, a humanized anti-PD-L1 antibody, had promising antitumor activity in patients with metastatic RCC^26^. The objective response rate reached 18% in those with PD-L1-positive staining of >1% of the tumor area, but dropped to 9% in those with PD-L1-positive staining of <1% of the tumor area. The objective response rate for patients with ISUP grade IV and/or sarcomatoid histology was 22%. For the patients who previously received antiangiogenic therapy, nivolumab also performed better than traditional second-line treatment of everolimus. In R.J. Motzer’s study, overall survival was longer and fewer grade 3 or 4 adverse events occurred with nivolumab than with everolimus. The objective response rate of the nivolumab reached to 25% which was greater than everolimus group which was only 5% (odds ratio, 5.98 [95% CI, 3.68 to 9.72]; P < 0.001)^27^. In our study, positive PD-L1 expression is indicative of a worse clinical outcome in Xp11.2 RCC patients. Prospective clinical trials are warranted to evaluate the treatment efficacy for Xp11.2 RCC patients, especially for those with advanced disease stage and a poor response to various therapeutic agents.

The current study had several limitations. First, its retrospective nature in a single institution may introduce selection bias. Prospective multicenter clinical trials are needed to validate the results. Second, although PD-L1 its expression status was closely associated with patient prognosis, the therapeutic effect of anti-PD-L1 mAbs in Xp11.2 RCC patients was not evaluated because these drugs are not yet available in China. Lastly, the dual color, break-apart FISH assay used in this study cannot detect each partner of the specific translocation, although it is an accurate and convenient diagnostic tool in formalin-fixed paraffin-embedded tissues.

In conclusion, positive PD-L1 expression is independently associated with tumor progression and predicts an adverse prognosis for Xp11.2 RCC patients. Importantly, our findings suggest that immunotherapy targeting the PD-1/PD-L1 pathway may represent a potential novel treatment for Xp11.2 RCC patients.

## Methods

### Patients and tissue samples

Tumor specimens from 2246 consecutive patients who underwent radical or partial nephrectomy to treat RCC from January 2008 to January 2015 at Fudan University Shanghai Cancer Center were analyzed. A total of 36 patients were confirmed to have Xp11.2 RCC using TFE3 break-apart FISH, as previously described^28^. Patient medical records were reviewed to collect demographic data, clinical manifestation, surgical techniques, pathological findings, clinical outcomes, and follow-up information. Tumor sizes were defined as the largest diameter of the surgically removed tumor mass. The 2010 American Joint Committee on Cancer TNM staging system was used to classify tumors. The study was carried out in accordance with the ethical standards of the Helsinki Declaration II and approved by the Institution Review Board of Fudan University Shanghai Cancer Center. Informed consent was obtained from each patient and the study was approved by our Institutional Ethics Committee.

### Immunohistochemistry staining

PD-L1 immunostaining of 36 Xp11.2 RCC tissue samples was performed using a rabbit monoclonal anti-PD-L1 antibody (1:150 dilution; Cell Signaling Technology, Billerica, MA, USA) and the Envision detection kit (Dako, Carpinteria, CA, USA). Tissue sections (4 μm thick) obtained from archived formalin-fixed, paraffin-embedded tissue blocks were deparaffinized in a xylene series and rehydrated with a graded ethanol series. Thereafter, endogenous peroxidase was quenched by incubation in 0.3% H_2_O_2_ for 15 min at 37 °C, and nonspecific binding was blocked by incubation in 10% normal goat serum for 60 min at room temperature. For antigen retrieval, sections were autoclaved in 0.01 M sodium citrate buffer, pH 6.0 (at 20 psi) for 10 min. Tissues sections were then incubated overnight with primary antibody at 4 °C. Chromogenic antigen detection was carried out using a peroxidase-conjugated secondary antibody (60 min incubation) and DAB reagents (1 min incubation; Envision detection kit, Dako, Carpinteria, CA, USA). Tissue sections were counterstained with Meyer’s hematoxylin (Thermo Fisher Scientific, Waltham, MA, USA).

All sections were examined and scored by two experienced pathologists blinded to all clinical data in an open discussion. The percentages of tumor cells with positive PD-L1 staining were quantified. In accordance with previous studies, PD-L1 tumor positivity was defined as membrane staining of ≥5% tumor cells^18, 23, 29^.

### Statistical analysis

To evaluate the relationship between the PD-L1 expression and clinicopathological parameters, the Student’s t-test and chi-square test were used for continuous and categorical variables, respectively. Univariate and multivariate Cox regression models were performed to evaluate the prognostic value of all parameters. Progression free survival (PFS) and overall survival (OS) for patients groups classified according to tumor PD-L1 expression level were calculated by the Kaplan–Meier method and compared using the log-rank test. All statistical tests were done using SPSS version 20 (SPSS Inc, Chicago, IL, USA). Two-tailed *p* values of <0.05 were considered statistically significant.
